# The impact of ribosome biogenesis in cancer: from proliferation to metastasis

**DOI:** 10.1093/narcan/zcae017

**Published:** 2024-04-15

**Authors:** Sseu-Pei Hwang, Catherine Denicourt

**Affiliations:** Department of Integrative Biology and Pharmacology, McGovern Medical School, The University of Texas Health Science Center, Houston, TX 77030, USA; The University of Texas MD Anderson Cancer Center UTHealth Houston Graduate School of Biomedical Sciences, Houston, TX 77030, USA; Department of Integrative Biology and Pharmacology, McGovern Medical School, The University of Texas Health Science Center, Houston, TX 77030, USA; The University of Texas MD Anderson Cancer Center UTHealth Houston Graduate School of Biomedical Sciences, Houston, TX 77030, USA

## Abstract

The dysregulation of ribosome biogenesis is a hallmark of cancer, facilitating the adaptation to altered translational demands essential for various aspects of tumor progression. This review explores the intricate interplay between ribosome biogenesis and cancer development, highlighting dynamic regulation orchestrated by key oncogenic signaling pathways. Recent studies reveal the multifaceted roles of ribosomes, extending beyond protein factories to include regulatory functions in mRNA translation. Dysregulated ribosome biogenesis not only hampers precise control of global protein production and proliferation but also influences processes such as the maintenance of stem cell-like properties and epithelial-mesenchymal transition, contributing to cancer progression. Interference with ribosome biogenesis, notably through RNA Pol I inhibition, elicits a stress response marked by nucleolar integrity loss, and subsequent G1-cell cycle arrest or cell death. These findings suggest that cancer cells may rely on heightened RNA Pol I transcription, rendering ribosomal RNA synthesis a potential therapeutic vulnerability. The review further explores targeting ribosome biogenesis vulnerabilities as a promising strategy to disrupt global ribosome production, presenting therapeutic opportunities for cancer treatment.

## Graphical Abstract

**Graphical Abstract:**
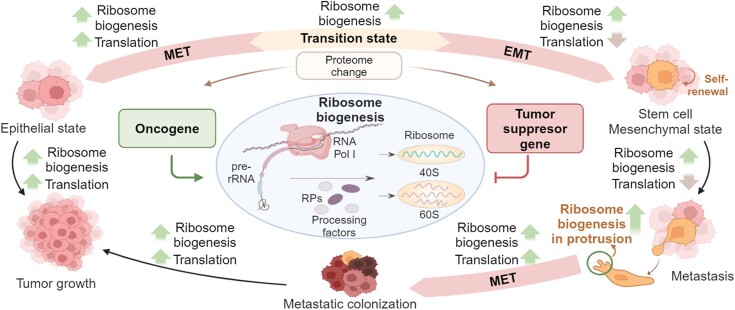

## Introduction

To meet the heightened metabolic demands inherent in tumorigenesis, cancer cells need to tightly regulate their protein synthesis capacity. Ribosomes, as key mediators of translation, often undergo increased production proportional to the rate of cell division (1). This upregulation in ribosome synthesis is a prevalent occurrence in various cancers (2–4). Numerous studies have established that this deregulation is not merely a consequence of hyperproliferation but a pivotal event in oncogenesis (5–8). The 80S eukaryotic translating ribosome is composed of the 40S and 60S subunits. The formation of the 40S subunit requires the coordinated synthesis of the 18S rRNA assembled with 33 ribosomal proteins, and the formation of the 60S subunit requires the coordinated synthesis of the 28S, 5.8S and 5S rRNAs joined by 47 ribosomal proteins (9,10). For each subunit, the rRNAs constitute the catalytic cores of the translation machinery, whereas the ribosomal proteins stabilize the structure and regulate the translation function of the ribosome.

**Figure 1.**
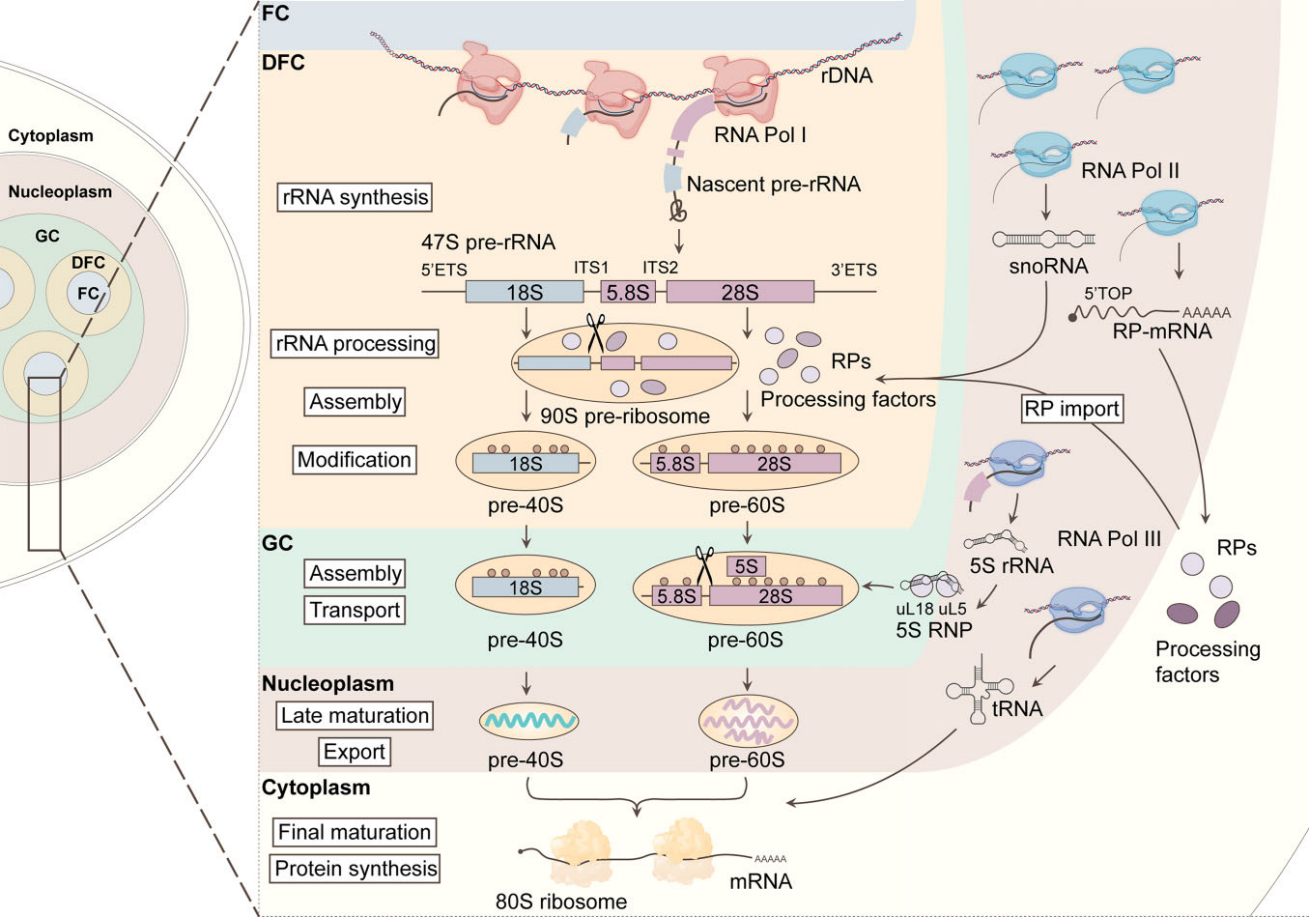
The different steps of ribosome biogenesis in the nucleolus. The process of ribosome biogenesis involves transcription by all three RNA polymerases. Initially, Pol I transcribes the 47S pre-rRNA in the nucleolus. This 47S pre-rRNA undergoes a series of cleavage and modification events orchestrated by various processing factors, resulting in the formation of the 18S, 5.8S and 28S rRNAs. These steps occur sequentially in distinct nucleolar subcompartments. Meanwhile, Pol III in the nucleoplasm transcribes the 5S rRNA, which complexes with uL18 and uL5 to form the 5S RNP, joining the pre-60S ribosomal subunit in the GC. RNA Pol II, also in the nucleoplasm, transcribes both snoRNAs and mRNAs encoding RPs and processing factors. These mRNA transcripts are translated in the cytoplasm, and their protein products are subsequently transported back to the nucleolus to participate in the assembly of the pre-40S and pre-60S subunits. Additionally, tRNAs, essential for translation, are transcribed by RNA Pol III. Throughout ribosome maturation, spanning various nucleolar subcompartments and nucleoplasm, the final maturation steps occur in the cytoplasm. This is also the site where protein synthesis takes place. Created with BioRender.com.

The biogenesis of these ribosomal subunits is a multi-step process initiated in the nucleolus with the transcription of the rDNA into a long precursor rRNA transcript of about 14 kb in size by RNA Pol I (47S pre-rRNA) (Figure 1). This 47S pre-rRNA transcript undergoes a series of coordinated maturation events involving the collaborative actions of over 200 trans-acting factors to generate the 18S, 5.8S and 28S rRNA molecules that will be assembled with ribosomal proteins (11–17). RNA Pol III produces the 5S rRNA that will be part of the 60S sub-unit (18). In this review, we will discuss the mechanisms used by cancer cells to hyperactivate rRNA synthesis to power the cellular translation machinery that supports tumorigenesis. We describe how oncogenes and tumor suppressor genes regulate rRNA transcription and processing and introduce new findings suggesting that increased ribosome biogenesis is critical for cancer stem cell-like maintenance, the epithelial-to-mesenchymal-transition (EMT) process, and metastasis. As most cancers depend on increased rates of ribosome biogenesis to sustain their metabolic demand, we review how this potential vulnerability can be exploited for the design of targeted cancer therapies.

## Aberrant rDNA transcription in cancer

The initial and rate-limiting step in ribosome biogenesis is the transcription of the pre-rRNA by RNA Pol I, whose regulation is intricately linked to internal and external signals that govern cell growth and division. These signals encompass nutrient availability, growth factors, and energy supplies, collectively influencing rRNA synthesis and all subsequent steps of ribosome production (19). The initiation of rRNA transcription is cyclic and coupled to cell-cycle progression (20). Upon receiving signals to divide and enter G1, cells upregulate rRNA synthesis and ribosome biogenesis to the rise in protein synthesis during the S-phase (20). The rate of rRNA synthesis peaks in the S and G2 phases but becomes repressed in mitosis before increasing again when cells re-enter G1 (20). Highly proliferative cancer cells, which constitutively activate signaling pathways promoting cell cycle progression, require intense protein translation to support their rapid growth. Consequently, the rate of rRNA synthesis is elevated in a broad spectrum of tumor types, and this phenomenon is regarded as a hallmark of human cancers (2,5,21,22).

Various mechanisms responsible for the elevated rRNA levels in cancers have been reported. In the following sections, we will describe how loss of tumor suppressor gene function and oncogenic signaling pathways feed into sustaining the hyperactivity of rRNA production. Furthermore, although it remains a relatively understudied area, we will discuss emerging genomic research suggesting that variations in rDNA copy number may be linked to cancer development (23).

### Basal RNA pol I transcription machinery

**Figure 2.**
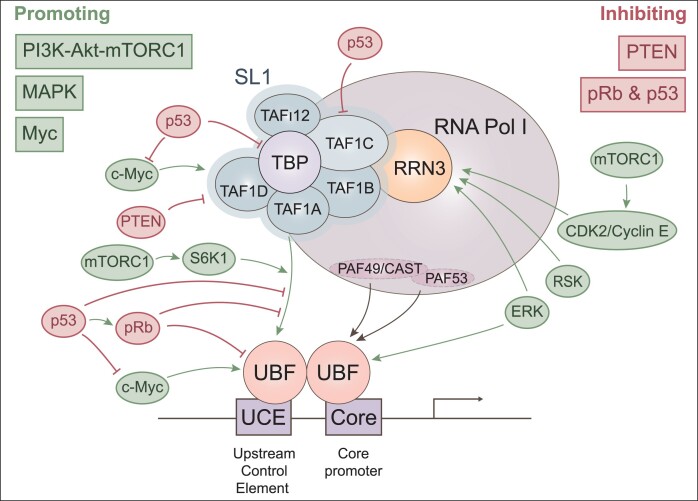
Signaling pathways regulate rRNA transcription by targeting RNA Pol I pre-initiation complex (PIC). A variety of oncogenes (green) and tumor suppressor proteins (red) regulate 47S rRNA transcription via activation or inhibition of the PIC of Pol I.

rDNA transcription necessitates the formation of the preinitiation complex (PIC), which assembly is initiated by the concerted binding of two Pol I–associated factors on the rDNA promoter: the upstream binding factor (UBF) and the promoter selectivity factor SL1 (TIF-IB in mice) (24) (Figure 2). SL1 is a multiprotein complex composed of the TATA-binding protein (TBP) and five TBP-associated factors (TAF1A, TAF1B, TAF1C, TAF1D and TAF_I_12) (25–28). SL1 is crucial for PIC formation as it recruits the initiation-competent form of Pol I (associated with RRN3 (analogous to mouse TIF-IA)) to the transcription start site, and it also stabilizes UBF at the rDNA promoter (29). UBF is part of the sequence-nonspecific class of high mobility group (HMG) proteins (30). Through multiple HMG boxes, UBF dimers promote bending and looping of 140 bp of rDNA promoter into a single turn, creating a nucleosome-like structure required to modulate rDNA transcription (31). UBF interacts cooperatively with SL1 at the rDNA promoter, through binding of its C-terminus to the TAF1A and TBP components of SL1 (32,33). UBF has been shown to be essential for PIC formation *in vivo*, and in addition to SL1, UBF also makes direct contact with the PAF53 and PAF49/CAST subunits of Pol I (34–36). As binding of UBF to the rDNA is not limited to the promoter but can be observed over the entire transcribed region, UBF is thought to also have a role in maintaining the overall euchromatic state of rDNA (37). RRN3 plays a crucial role in recruiting Pol I to the UBF/SL1 complex at the rDNA promoter (29). By associating with a small fraction of Pol I complex, accounting for around 10%, RRN3 facilitates the precise positioning of Pol I at the rDNA by making contact with two subunits of SL1 (29). With their central roles in regulating Pol I activity, UBF, SL1 and RRN3 are the main targets of tumor suppressors and oncogenic signaling pathways controlling rRNA transcription in cancers, a topic further explored in the subsequent discussion.

### Synthesis of 5S rRNA by RNA Pol III

Unlike the synthesis of the 18S, 5.8S and 28S rRNAs, the 5S rRNA is transcribed by RNA Pol III. In humans, the 5S rRNA genes are arranged in tandem repeats located uniquely on chromosome 1 (38,39). Interestingly, this region of chromosome 1 containing the *5S rRNA* genes is positioned in close proximity to nucleoli (40). Most RNA Pol III transcribed genes have been shown to co-localize to nucleoli and it is hypothesized that this spatial regulation is important for the coordinated transcription of all RNA components of the translational machinery (41). Transcription of the pre-5S rRNA by RNA Pol III requires the specific regulatory transcription factor IIIA (TFIIIA) that forms a complex with the general class III initiation factors TFIIIB and TFIIIC on the 5S gene promoter (42,43). The transcribed precursor contains a 3′ uridine-rich extension that is recognized by the La protein, which functions as a chaperone important for the stability and maturation of 5S (44). Following 3′ end processing by the REX1, REX2 and REX3 exonucleases, the 5S rRNA associates with the ribosomal protein uL18 (formerly RPL5) then moves to the nucleolus, where joined by ribosomal protein uL5 (formerly RPL11) is integrated into the maturing pre-60S ribosome (45,46). uL18, together with uL5 and the 5S rRNA, constitute the 5S ribonucleoprotein particle (5S RNP), which has ribosome-independent functions in growth and cell cycle regulation, acting as a central regulator of p53 stability and activation (47–49).

### Mechanisms responsible for increased rRNA synthesis in cancers

Although gain of function mutations affecting the genes coding for RNA Pol I and the regulatory factors described above are rarely observed in most cancers, their activity is consistently found upregulated in tumor cells (50–52). In most cases, increased rRNA synthesis results from either gene overexpression of the basal components of the Pol I machinery or loss of tumor suppressor function and hyperactivation of signaling pathways regulating their activity (22,53).

### Overexpression of the Pol I transcriptional machinery components in cancers

The expression of most genes encoding basal components of the Pol I transcriptional machinery, including Pol I subunits, are consistently found overexpressed in a wide array of cancer types (3,50–52,54). UBF expression is specifically elevated in hepatocellular carcinoma as well as in colorectal cancer and increased rates of rRNA synthesis associated with high UBF levels are commonly observed in rapidly proliferating tumors (3,54). Ectopic overexpression of UBF is sufficient to promote rRNA synthesis and cell proliferation of human cells, whereas its depletion suppresses cell proliferation and induces cell death (54,55). Several datasets from The Cancer Genome Atlas (TCGA) revealed that the expression of most components of the Pol I transcriptional machinery is increased in invasive breast cancer, with RRN3 and the SL1 subunit TAF1A showing the highest percentage of upregulation (52,56). Notably, patients who presented elevated expression of at least one of the basal Pol I transcription machinery genes had a significantly poorer prognosis. Interestingly, RRN3 and TAF1A also showed gene amplification in early breast cancer lesions, such as non-invasive ductal carcinoma *in situ* (DCIS) and atypical ductal hyperplasia (ADH). Accordingly, ectopic overexpression of RRN3 in human mammary epithelial cells (HMECs) is sufficient to increase rRNA transcription and induce morphological changes reminiscent of DCIS (52). This observation, coupled with the identification of heightened expression of the core Pol I transcription machinery in the early stages of breast cancers, lends credence to the intriguing hypothesis that increased rRNA synthesis could play a role in initiating breast cancer. While the overexpression of the basal Pol I transcription machinery seems sufficient to elevate rRNA transcription and induce cellular changes linked to malignancy, it is yet to be investigated whether their expression alone is enough to drive tumorigenesis using appropriate *in vivo* cancer models.

### Oncogenic signaling pathways control rRNA synthesis in cancers

Most signaling pathways controlling cell growth and proliferation converge to regulate different steps of rRNA synthesis (Figure 2). In normal cells, mitogens, growth factors, and nutrient availability stimulate rRNA transcription and processing, whereas anti-mitogen signaling, nutrient deprivation, stress, and DNA damage have a repressive effect on Pol I activity and rRNA synthesis in general (57, 19, 58–62). With their acquired self-sufficiency for growth factors and insensitivity to anti-growth signals, cancer cells have a multitude of hyperactivated signaling pathways and protein kinases that directly stimulate Pol I complex components and rRNA processing factors. On the other hand, several tumor suppressor proteins that repress Pol I transcription are frequently lost in tumors and thus represent another mechanism by which cancer cells upregulate rRNA production. In general, most signals primarily converge on the three key basal Pol I components: UBF, SL1 complex and RRN3. Multiple kinases concurrently target these factors, indicating that the concomitant activation of UBF, SL1 and RRN3 contributes to both robust signal transduction and the precise adjustment of Pol I activity.

### PI3K–Akt–mTORC1

The mechanistic target of rapamycin (mTOR) serine/threonine kinase is considered to be one of the master regulators of ribosome biogenesis and mRNA translation (63,64). Several proteins are associated with mTOR, and distinct components define two mTOR protein complexes, mTOR Complex1 (mTORC1) and mTORC2 (65). mTORC1 receives, integrates and transduces growth signals in response to the availability of nutrient and growth factors (64). mTORC1 is predominantly recognized for its role in promoting protein synthesis through direct activation of ribosomal protein S6 kinase 1 (S6K1) and the eukaryotic initiation factor 4E binding protein 1 (4E-BP1) required for translation initiation (64). Moreover, mTORC1, by deactivating 4E-BP proteins, facilitates the translation of a specific subset of mRNAs characterized by 5′ terminal oligopyrimidine (TOP) motifs or TOP-like motifs, including mRNAs encoding ribosomal proteins (66). mTORC1 was also shown to bind to rDNA genes and indirectly activate Pol I transcription mainly by regulating the activity of the transcription factor RRN3 via activation of CDK/Cyclin E (58). mTORC1 also increases UBF interaction with SL1 on rDNA by stimulating the phosphorylation of UBF C-terminal domain by S6K1 (67). Although the mechanism is not very clear, mTORC1 activity is required for the events required to generate the mature 18S and 28S rRNA (68). The synthesis of the 5S rRNA is also under the regulation of mTORC1, where mTOR has been shown to associate with general class III initiation factor TFIIIC on 5S rRNA genes and phosphorylate MAF-1, a repressor that binds and inhibits Pol III transcription (69,70). Thus, mTORC1 promotes all aspects of ribosome biogenesis, from the synthesis of ribosomal proteins to transcription and processing of pre-rRNA as well as the synthesis of the 5S rRNA by RNA pol III (71). Inhibition of mTORC1 signaling by rapamycin impairs rRNA synthesis, implying the essential role of mTORC1 in ribosome biogenesis. This process likely occurs concurrently with cytoplasmic translational regulation, enabling cancer cells to optimize translational capacity during tumorigenesis.

### Mitogen-activated protein kinases (MAPKs) cascade

Mitogen-activated protein kinase (MAPK) cascades are central signaling pathways involved in regulating cell proliferation and survival (72,73). One of the best-studied mammalian MAPK pathways is the Raf–MEK–ERK pathway, which is constitutively activated in a large proportion of human cancers (74). Not surprisingly, this pathway has been shown to promote a significant increase in rRNA synthesis rapidly after growth factor stimulation, mainly through regulation of the two basal Pol I transcription factors, RRN3 and UBF (75,76). Both ERK and RSK phosphorylate RRN3 at two serine residues (ser633 and ser649), which were shown to be required for its activation and the initiation of pre-rRNA synthesis (76). Additionally, epidermal growth factor (EGF)-dependent activation of ERK1/2 results in phosphorylation of UBF at two threonine residues (Thr117 and Thr201) that are critical to mediate Pol I transcription elongation (75). MAP kinase signaling also regulates mTORC1 activation, thus contributing two-fold to upregulating ribosome biogenesis (72,73).

More recently, increased ERK1/2 activity induced by oncogenic RAS has emerged as an important regulator of Nucleolin's functions (77). Nucleolin, an rRNA-binding protein involved in various stages of ribosome biogenesis, was shown to be an indirect phosphorylation target of ERK1/2 in a mouse model of oncogenic RAS-driven pancreatic ductal adenocarcinoma (PDAC). Under the influence of oncogenic RAS, this phosphorylation, most likely performed by active CK2, enhances Nucleolin binding affinity to preribosomal-RNA, amplifying rRNA synthesis, ribosome biogenesis, and mRNA translation. Notably, this upregulation of ribosome biogenesis facilitated by Nucleolin was shown to be indispensable for the proliferation and tumorigenesis of PDAC cells. These findings underscore the pivotal role of oncogenic RAS signaling in governing rRNA synthesis and ribosome biogenesis in the context of pancreatic cancer (77).

### Myc

The transcription factor c-Myc is one of the most frequently activated oncogenes in human cancers (78). Its growth-promoting capacity relies primarily on increasing ribosome biogenesis and mRNA translation (79). The effect of c-Myc in stimulating ribosome biogenesis is threefold as it regulates RNA pol I, pol II and pol III, respectively involved in the synthesis of 18S/5.8S/28S rRNAs, ribosomal proteins and the 5S rRNA (80,81). In addition, c-Myc stimulates different steps of rRNA processing by directly controlling the expression of diverse ribosome assembly factors, exonucleases, rRNA-modifying enzymes and the snoRNAs required to guide rRNA modifications (82). In addition to stimulating the expression of UBF by Pol II transcription, c-Myc also localizes to the nucleolus where it directly binds to E-box elements present on the promoter of rDNA genes, whereby promoting rRNA synthesis by recruiting SL1 for the assembly of the RNA Pol I pre-initiation complex (83). Significantly, in mouse models, specifically inhibiting Pol I transcription effectively slowed the advancement of B-cell tumors induced by Myc overexpression through the induction of p53-dependent apoptosis (7). MYC’s ability to increase rRNA synthesis is thus necessary for its oncogenic potential (6), emphasizing the concept that increased rRNA production is not just a mere consequence of rapid proliferation but is a requirement for tumor progression.

### Tumor suppressors inhibit rRNA synthesis

#### pRb and p53

The retinoblastoma protein (pRb) and p53 are both tumor suppressors that negatively regulate cell cycle progression by controlling major checkpoints that block cell division in response to growth inhibitory or stress signals (reviewed in (84,85)). The functions of pRb and p53 are compromised in a high percentage of cancers either due to inactivating gene mutations or hyperactivation of oncogenic pathways disabling their functions (84,85). In cells induced to differentiate, pRB accumulates in nucleoli concomitantly with inhibition of rRNA synthesis, suggesting a role for pRb in Pol I repression (86). Initially, it was shown that pRb has the capability to suppress rRNA synthesis by binding to UBF and impeding the formation of the Pol I transcription complex (86). The exact mechanism by which pRb negatively affects UBF function remains controversial as later studies have proposed that pRB impairs UBF association on rDNA promoter (87,88), whereas others have shown that both pRb and p130 (a member of the Retinoblastoma family of tumor suppressor genes) disrupt the interaction between UBF and SL1 (89). Nevertheless, repression of rRNA synthesis by inactivation of UBF is likely to be an important mechanism by which pRb exerts its tumor suppressor functions. p53 has been shown to repress rRNA synthesis by inhibiting both Pol I and Pol III and mutations that affect its cell cycle inhibitory functions also impair its ability to inhibit rRNA synthesis (57,90). p53 hinders Pol I transcription by directly engaging with TBP and the TAF1C components of SL1. This interaction disrupts the recruitment of UBF to the Pol I pre-initiation complex on rDNA promoters. Additionally, p53 indirectly disrupts UBF activity by inducing the cyclin-dependent kinase (CDK) inhibitor p21^Cip1^, thereby preventing the inactivation of pRb by cyclin–CDK complexes (57,89). In human cancers, nucleolar sizes are often correlated to pRb and p53 status, such that cancers with impaired Rb function and mutated p53 display higher rates of rRNA synthesis and are generally more aggressive (91).

#### PTEN

The phosphatase and tensin homolog deleted on chromosome ten (PTEN) is a tumor suppressor whose functions are frequently lost in brain, breast, prostate, pancreas and ovarian cancers (92). PTEN regulates a variety of cellular processes, such as proliferation, cell survival, and maintenance of genomic integrity (93). PTEN mediates these functions in part by downregulating the activity of the PI3K-Akt and mTORC1 axes, which both promote rRNA synthesis, cell growth and proliferation as described above (reviewed in (94)). PTEN has been shown to inhibit rRNA synthesis by reducing the occupancy of SL1 from rDNA promoter, a function that requires its lipid phosphatase activity (95). In addition, in a complex with Glycogen synthase kinase 3 beta (GSK3β), PTEN can be found on the promoter and coding regions of rDNA genes. Constitutively active GSK3β abolishes rRNA synthesis, whereas inhibition of its activity upregulates rRNA transcription and cell proliferation (96). More recently, a newly identified N-terminal extended isoform of PTEN, known as PTEN-β, has been observed to primarily reside in the nucleolus. This isoform physically interacts with nucleolin, a factor involved in multiple steps of ribosome biogenesis, to promote its inactivation by dephosphorylation. Consequently, this interaction negatively regulates both ribosomal DNA transcription and ribosomal biogenesis (97).

## Aberrant rRNA processing in cancer

The maturation of the 47S pre-rRNA is co-transcriptional and is initiated as soon as the transcript emerges from the Pol I complex by the assembly of ribosome biogenesis factors and ribosomal proteins into a premature ribosomal particle in the nucleolus (12,98,99). The earliest stable pre-ribosomal intermediate to form is called the 90S pre-ribosome or small subunit processome (SSU), which comprises >70 assembly factors, including the transcription U three proteins (t-UTPs), the U3 small nucleolar ribonucleoprotein (U3 snoRNP), several small subunit ribosomal proteins and the nascent pre-rRNA transcript. These factors act as scaffolds and chaperones for the nascent pre-rRNA, ensuring correct conformation and coordination of the maturation process (11,100–103). In the subsequent maturation steps, a series of coordinated endo- and exonucleolytic cleavages take place to remove the spacer regions (ETS and ITS). Endonucleolytic cleavage at specific sites within the 5′-ETS and the ITS1 is key to the transcript separating into a pre-40S subunit with the 18S rRNA and a longer pre-rRNA that contains both the 28S and 5.8S rRNA (11,12,16,100,103–105).

Following separation, the pre-40S subunit undergoes maturation steps to refine its head region and adjust the conformation of helices in the 18S rRNA. Several factors participating in these steps are associated with the pre-40S subunit in the earlier stage and continue to remain linked to the pre-40S subunit in the cytoplasm. For a recent review on 40S biogenesis we refer to (102,103,106,107). The pre-60S particle containing the 32S pre‐rRNA—comprising both the 5.8S and 28S rRNA regions—undergoes its independent maturation processes simultaneously (102,103,105,108). Initial pre‐60S rRNA processing involves folding, compaction, cleavages, and the concurrent incorporation of ribosomal proteins, orchestrated by various biogenesis factors including nucleases, ATP‐dependent RNA helicases, AAA‐ATPases and GTPases. Different domains of 28S rRNA and 5.8S rRNA fold and position themselves for the progressive formation of the peptidyl transferase center (PTC), polypeptide exit tunnel (PET), and the 40S/60S subunit interface (11,12,103,105). As mentioned earlier, the 5S RNP is transported into the nucleus by the chaperone Syo1/HEATR3 to assemble with the pre-60S subunit in the nucleolus, a process facilitated by the Rpf2‐Rrs1 complex. The 5S RNP constitutes the central protuberance, which undergoes a final rotation step in subsequent maturation stages (103,109–113).

Pre‐rRNA processing during 60S maturation begins with removal of the remaining ITS1 spacer at the 5′ end of 32S pre‐rRNAs (12,103). Subsequently, cleavage in the ITS2 region mediated by the endonuclease RNase PNK complex (LAS1L-NOL9)) separates the pre-5.8S and pre-28S rRNAs (103,114–117). The transition of pre-60S particles from the nucleolus to the nucleoplasm involves significant alterations in both structure and composition, including the release of numerous accessory factors and recruitment of new ones. Notably, the Rix1 complex (Pelp1, WDR18 and TEX10 in human) plays a crucial role in initiating the formation of the central protuberance. It recruits the ATPase MDN1, which catalyzes the release of the Rpf2-Rrs1 complex, leading to the 180° rotation of the 5S RNP and concurrent maturation of the PET and PTC (103,113). The Rix complex within the rixosome represents an interaction hub that directly coordinate the 5S RNP rotation with the recruitment of the endonuclease RNase PNK to initiate the cleavage in ITS2, which will be subsequently processed by the exosome complex (105). Eventually, both pre-40S and pre-60S subunits, accompanied by numerous factors, are exported to the cytosol for the final maturation steps, rendering them fully functional for translation (reviewed in (103,118)). For a comprehensive understanding of rRNA processing and ribosome assembly, we refer to recent reviews that offer detailed insights into this complex process (11,12,103).

Throughout the co-transcriptional maturation process outlined above, the rRNAs undergo extensive chemical modifications mediated by small nucleolar ribonucleoproteins (snoRNPs) (119–121). snoRNPs are composed of structural and enzymatic proteins combined with noncoding RNA molecules, called small nucleolar RNAs (snoRNAs), that guide the enzymatic complex on the nucleotide that will be modified (119–121). Among all the chemical modifications found on rRNA, pseudouridylation (Ψ) and ribose methylation (2′-O-me) are the most frequent (122). Two distinct types of snoRNPs responsible for these rRNA modifications have been described: H/ACA box and C/D box snoRNPs, which respectively guide Ψ and 2′-O-me (119–121). Expression of snoRNPs, including both snoRNAs and their enzymatic protein components, is found to be frequently deregulated in multiple cancers (123,124). Interestingly, the DEAD-box RNA helicase DDX21, also overexpressed in several cancers, appears to be a crucial player in coordinating Pol I transcription with concomitant rRNA modifications (125–128). In the nucleolus, DDX21 associates with actively transcribed rDNA genes and directly contacts nascent rRNA and snoRNAs to promote co-transcriptional rRNA processing and modifications (129). Aberrant patterns of rRNA modification have been observed on cancer ribosomes, and these changes have been associated with a decrease in translation fidelity and an increase in the translation of oncogenic mRNAs (130–135) This topic is explored further in the review from Jean-Jaques Diaz in this collection.

Compared to the extensive studies on rRNA processing factors in yeast, parallel functional investigations into these factors in humans are relatively limited. Although many of the factors are conserved from yeast to human, accumulating efforts in wide-scale screening and proteomic analyses have identified an increasing number of factors participating in human ribosome biogenesis process, suggesting more intricate regulatory pathways involved in human ribosome biogenesis compared to yeast (14–17,136–139). The overexpression of factors involved in different steps of ribosome biogenesis have been reported in various types of cancers. Recent pan-cancer analyses have further revealed the overall upregulation of genes involved in ribosome biogenesis across various types of human cancer, which is correlated with poor survival and prognosis for patients (140). Together, these imply that rRNA processing factors may have potential roles in promoting cancer development through ribosome biogenesis regulation. However, their specific functions in human ribosome biogenesis and how this contributes to cancer remain understudied. This area merits increased attention, particularly for exploring their potential as therapeutic targets. Here, we discuss some examples in recent studies that illustrate the overexpression of rRNA processing factors in cancer and explore their potential mechanisms.

An example is the aberrant overexpression of ZCCHC7 in lymphoma cells. ZCCHC7 (homolog of Air2p in yeast) is a potential component of the TRAMP-like complex, interacting with the exosome complex, which is the exonuclease complex essential for pre-rRNA processing (141–144). In lymphoma cells, the overexpression of ZCCHC7 disrupts the processing of 5.8S rRNA, leading to an accumulation of the precursor 5.8S + 40 rRNA (145). While the mechanism remains to be fully elucidated, it was hypothesized that the overexpression of ZCCHC7 may negatively regulate the nuclear RNA exosome subunit EXOSC10, which is responsible for trimming the 3′ end of the 5.8S + 40 rRNA, ultimately producing the mature 5.8S rRNA. In addition to the processing defects, analysis of nascent peptide synthesis indicates an alteration in protein synthesis under ZCCHC7 overexpression in lymphoma cells. Specifically, the translation of a subset of oncogenes is promoted, while the translation of several tumor suppressors is inhibited. The observed alterations in the lymphoma proteome might stem from the emergence of a ribosome subpopulation due to shifts in the kinetics of 5.8S rRNA processing. However, further investigations are necessary to precisely delineate how these changes influence the translation capacity of ribosomes (145).

Another instance involved is PELP1, a component in the Rix1 complex, identified as a proto-oncogene overexpressed in various cancer types, including breast, endometrial, ovarian, salivary, prostate, lung, pancreas, colon, and gastric cancer (146–148). PELP1 overexpression correlates with poor patient outcomes and has been implicated in promoting metastasis and therapeutic resistance (146,149–152). While PELP1 is recognized as a coactivator for various steroid receptors, including estrogen receptors and androgen receptors, recent cryo-EM structural analysis indicates that the region required for binding to steroid receptors is inaccessible when PELP1 is associated with WDR18 in the Rix1 complex (153). This implies that PELP1's role in ribosome biogenesis within the Rix1 complex operates independently of its activity in regulating steroid receptor coactivation (153). Furthermore, in addition to its involvement in rRNA processing, PELP1 is recruited to rDNA promoters to facilitate rRNA transcription, suggesting its dual roles in promoting ribosome biogenesis (154). Interestingly, other components of the Rix1 complex (WDR18 and TEX10) are also found overexpressed in cancer, indicating that the function of this complex is required to support high rates of ribosome biogenesis in cancer (140,155,156). While there are clear indications of PELP1's involvement in cancer proliferation and progression, its precise impact on cancer development via ribosome biogenesis, rather than hormone receptor activity regulation, remains largely unexplored. The pivotal role of PELP1 in cancer has prompted the development of specific inhibitors, as discussed in subsequent sections.

### Other pathways regulating rRNA synthesis in cancers

A recent study has uncovered a critical link between the reactivation of RNA Pol I and the survival of breast cancer cells post-radiotherapy (157). In irradiated tumor cells, the hedgehog pathway transcription factor GLI1 becomes activated and translocates to the nucleolus through the treacle ribosome biogenesis factor 1 (TCOF1). This translocation is a crucial step as GLI1, by regulating histone acetylation at the RNA Pol I promoter through a possible interaction with the N-acetyltransferase 10 (NAT10), facilitates the reactivation of rRNA synthesis in response to radiation. The resurgence of RNA Pol I activity, in turn, amplifies resistance to radiotherapy, promoting the survival of cancer cells and laying the groundwork for recurrence and metastasis. These findings highlight the significance of hedgehog pathway dysregulation in driving rRNA synthesis, a factor contributing to the recurrence of cancer. Moreover, they shed light on a potential strategic avenue—inhibiting RNA Polymerase I following radiotherapy—as a means to mitigate cancer recurrence (157).

An emerging body of studies has revealed that an additional level of regulation over rDNA transcription is wielded by non-coding RNA species, specifically long non-coding RNAs (lncRNAs) (158). An illustration of how lncRNAs impact the production of rRNA can be observed in the case of the lncRNA Promoter And Pre-rRNA AntiSense (PAPAS). PAPAS levels increase during periods of quiescence (159) and hypotonic stress (160). In quiescence, PAPAS recruits the Suv4-20h2 histone methyltransferase, promoting the trimethylation of histone H4 at lysine 20 (H4K20me3) and consequently instigating the transcriptional silencing of rRNA genes (159). Similarly, under conditions of hypotonic stress, when Suv4-20h2 undergoes degradation, PAPAS induces nucleosome repositioning through the NuRD chromatin remodeling complex, once again leading to the silencing of rDNA (160). PAPAS is found downregulated in human breast cancers. Consistent with its role in repressing Pol I activity, PAPAS has been found to promote the lactogenic differentiation of mammary epithelial cells and inhibit the tumorigenesis and progression of breast cancer (161).

Another notable instance of a lncRNA regulating rRNA transcription is exemplified by SLERT, a lncRNA containing box H/ACA small nucleolar RNA (snoRNA) sequences at both ends (127). Expressed in embryonic stem cells and various human cancer cell lines, SLERT relies on the H/ACA snoRNAs for accurate processing and localization to the nucleoli. In terms of mechanisms, SLERT forms a complex with the DEAD-box RNA helicase DDX21 through its non-snoRNA region. While DDX21 plays a crucial role in effective rRNA processing, it adopts ring-shaped structures around Pol I complexes, concurrently hindering pre-rRNA transcription. SLERT binding induces allosteric changes in individual DDX21 molecules, disrupting the integrity of the DDX21 ring and relieving its suppression on Pol I transcription. The formation of DDX21 rings in this process could potentially synchronize pre-rRNA transcription and modification, leading to increased efficiency in pre-rRNA processing. Significantly, studies have demonstrated that inhibiting SLERT reduces tumorigenic potential in both *in vitro* and *in vivo* xenograft models (127). Therefore, targeting SLERT or disrupting the interaction between SLERT and DDX21 emerges as promising strategies to hinder rRNA synthesis for anti-cancer treatment.

One mechanism contributing to the increased expression of RNA Pol I machinery components in cancer involves their post-transcriptional regulation by microRNAs (miRNAs). Specifically, miR-330-5p and miR-1270 target the 3′UTR of key RNA Pol I core components, including TAF1A, TAF1B, TAF1C, RRN3 and POLR1B, leading to their downregulation (162). In various cancers, such as lung adenocarcinomas, a notable reduction in the expression of these miRNAs results in the overexpression of Pol I machinery components. The ectopic overexpression of miR-330-5p and miR-1270 demonstrates a suppressive effect on rRNA synthesis and enhances sensitivity to chemotherapeutic drugs, leading to reduced tumor growth in a mouse xenograft model (162). This unveils a potential therapeutic avenue, suggesting that strategies focused on delivering these miRNAs *in vivo* could be explored for treating cancers characterized by high Pol I activity.

The long non-coding RNA SAMMSON, a survival oncogene overexpressed in a considerable portion of skin and uveal melanomas, has recently been implicated in the synthesis of both mitochondrial and nuclear-encoded rRNAs (163–165). Specifically, it was initially shown to facilitate the efficient mitochondrial targeting of p32, which is necessary for the maturation of mitochondrial 16S rRNA (165). Furthermore, subsequent studies have also revealed SAMMSON's involvement in regulating the subcellular localization of the 5′-3′ exoribonuclease XRN2 to the nucleolus, where it acts to ensure the proper maturation of the 5.8S and 28S rRNAs. Mechanistically, by binding to CARF, which typically sequesters XRN2 in the nucleoplasm, SAMMSON promotes the localization of XRN2 in the nucleolus, fostering efficient rRNA maturation. Consequently, SAMMSON supports both cytosolic and mitochondrial protein synthesis, thereby promoting cancer growth (165).

### rDNA gene variation associated with cancer

The inherent repetitiveness of ribosomal DNA (rDNA) genes, coupled with their distinction as the most transcribed region in the human genome, makes them susceptible to recombination and genomic instability, potentially contributing to the development of diseases. While the precise role of rDNA instability in driving oncogenic transformation remains uncertain, studies have reported an association between the two. Notably, rDNA stands out as one of the most variable regions in the human genome concerning copy number and sequence alterations, with computational analyses revealing changes in both aspects in human cancer genomes.

Intriguingly, certain cancers exhibit higher rDNA copy numbers, while others display lower rDNA copy numbers, particularly those associated with elevated mTOR activity (23). Surprisingly, in the Pten^-/-^ leukemia mouse model characterized by heightened mTORC1 activity, cells with lower rDNA copies display heightened sensitivity to DNA damage, while showing increased rDNA transcription associated with rapid proliferation and elevated protein synthesis—a phenomenon contrary to the expectation that increased rRNA synthesis correlates with higher rDNA copy numbers (23). One potential explanation for this paradox is that the loss of PTEN results in the accumulation of double-strand breaks (DSBs) due to replication stress. The highly repetitive and heavily transcribed nature of rDNA makes it a fragile region within the human genome, heightening the risk of instability that could potentially lead to cancer. In this context, the reduction of rDNA may favor rapid cell cycle progression, as rDNA replication is energetically demanding, suggesting that cancer employs strategies to adapt to the loss of rDNA copies without compromising rRNA production (23). While investigations involving large populations are essential to elucidate the associations between rDNA copy number and cancer development and to evaluate the potential of rDNA copy number as a predictive marker in diagnosis, the current body of research implies that variations in rDNA may represent a common characteristic of cancer that may influence ribosome biogenesis.

Exploring the molecular mechanisms connecting rDNA copy number variation to cancer, a study on ALT (Alternative Lengthening of Telomeres)-positive tumors uncovered a potential mechanism related to alterations in histone modification patterns (166). Loss of rDNA, observed in ALT-positive tumors associated with mutations in the ATRX gene, interferes with the assembly of heterochromatin at rDNA repeats, diminishing the integrity of rDNA chromatin and resulting in decreased rRNA synthesis. Cells lacking ATRX also show increased susceptibility to RNA Pol I inhibition, such as CX-5461 treatment, indicating a connection between rDNA copy number, mechanisms preserving genome stability and regulation of rDNA transcription (166). As cancers heavily rely on heightened ribosome biogenesis, the amplification of rDNA supports increased rDNA transcription. Previous studies suggest that an abundance of rDNA copies is advantageous in preserving genome integrity, with stably silenced heterochromatic rDNA genes contributing to promoting genome stability. Conversely, the strategic loss of rDNA copy number may be selected to conserve energy during rapid replication, potentially contributing to increased sensitivity to damage and genomic instability, facilitating subsequent mutagenesis and adaptation in the context of cancer (167). In summary, these findings indicate that the variation in rDNA copy number could function both as a driver and a consequence of cancer. The interplay observed between changes in copy number and transcriptional activity highlights a dynamic balancing act that can be exploited in cancer progression.

## Ribosomopathies and cancer

Ribosomopathies constitute a large and growing collection of disorders resulting from an abnormal reduction in ribosome production, leading to disruptions in protein synthesis (for recent reviews, see (168–170)). The low production of ribosomes can result from abnormalities in ribosome biogenesis at various stages, spanning from the disruptions in rRNA transcription from Pol I machinery (171), rRNA processing factors including modifications and cleavage (172–174) (for a review, see (170)), and the assembly of ribosomes (175), including inactivating mutations in ribosomal proteins (176).

Ribosomopathies present a complex disease progression. Initially, symptoms like bone marrow failure, craniofacial or skeletal defects, and anemia arise due to cellular hypoproliferation (176). Yet, with proper therapy, individuals may transition to a hyperproliferative state, increasing the risk of cancer in later stages (177). This prompts intriguing questions about the connection between the initial hypoproliferative phenotype and later cancer predisposition, with various mechanisms proposed across ribosomopathies. One possible mechanism involves altered mRNA translation programs by specialized onco-ribosomes (see review (169)). Supporting evidence suggests that alterations in ribosomal proteins and biogenesis factors can change ribosome composition, creating specialized ribosomes that can reshape translational landscapes (178–181). Ribosomal defects in ribosomopathies have been linked to translation reprogramming, notably impacting the translation of crucial hematopoietic regulator mRNAs (182,183). While precise molecular mechanisms are yet to be fully elucidated, this alteration in translational programs may set the stage for a pre-oncogenic state, shifting the cellular balance from hypo- to hyperproliferation. Studies investigating the functions of mutant ribosomal proteins have revealed changes in translation speed and fidelity, including near-cognate amino acid misincorporation and stop-codon read-through (184,185), which could also be a mechanism at play in establishing a pre-oncogenic proteome. Moreover, ribosome deregulation can lead to disruptions in metabolic homeostasis and stress responses, such as elevating reactive oxygen species (ROS), contributing to genome instability and secondary mutations giving growth advantages (169,186,187). Another potential mechanism proposed involves the acquired p53 loss of function mutation, driven by selective pressure as a survival strategy of cells in ribosomopathies (168,170).

In light of these complex interactions between ribosomal defects and cancer development, further elucidating the molecular mechanisms underlying the transition from hypoproliferation to cancer is essential. The emerging concept of onco-ribosomes and specialized translation in cancer research presents intriguing avenues for exploration. Therefore, determining ribosome composition through multi-approach proteomics, cryo-EM structural analysis, and characterization of rRNA modification patterns is crucial. Additionally, deeper analysis of their regulation of translational programs will likely identify novel therapeutic targets for ribosomopathies and cancer.

## Ribosome biogenesis in cancer stem cells, EMT and metastasis

Recent studies have highlighted the pivotal role of ribosome biogenesis not only in cell proliferation but also in key aspects of cancer progression, including Epithelial–Mesenchymal Transition (EMT), cancer stemness, and metastasis. While it is widely acknowledged that rapidly dividing cells enhance their ribosome biogenesis to meet the demand for high protein synthesis accompanying rapid growth, this is not universally applicable. For instance, stem cells, which have a slower proliferation rate, exhibit elevated ribosome biogenesis despite maintaining low global rates of protein synthesis relative to the differentiated cell types they give rise to (188–191). These mechanisms, crucial for self-renewal and differentiation, may share similar functions in the tumorigenesis process. Consequently, the aggressiveness of a tumor is prominently reflected in its stage of differentiation, with poorly differentiated tumor cells possessing stem cell-like characteristics often exhibiting more aggressive behavior and increased susceptibility to invasion and metastasis (192,193). In this context, we discuss recent research suggesting that certain tumor cells employ comparable mechanisms of ribosome biogenesis and translation control to those observed in stem cells, thereby sustaining essential plasticity characteristics crucial for metastasis.

There is a close association between the rate of ribosome biogenesis and cell fate, regulating cellular plasticity, differentiation status, and the acquisition of stem cell-like properties (194,195). A high rate of ribosome biogenesis is a characteristic feature of stem cells that sustains self-renewal ability and plasticity (188,189). Studies in hematopoietic stem cells (HSCs) have associated the decline in rRNA synthesis with the aging of these cells. As a result, stem cells lose their regenerative potential gradually, suggesting that maintaining a high rate of ribosome biogenesis is crucial for their survival (196,197). Despite having a high level of ribosome biogenesis, stem cells surprisingly maintain a lower translation level compared to differentiated cell types. (190). This seemingly contradictory phenomenon is postulated to result from stem cells maintaining a high level of latent ribosomes, preparing for the rapid protein synthesis upon differentiation signals, thereby establishing a new proteome promptly (191, 198, 199). Stem cells increase their ribosome biogenesis by expressing components and factors involved in RNA Pol I and Pol II machinery. This promotes the synthesis of rRNA, ribosomal proteins, and factors that are responsible for rRNA processing and ribosome maturation (189,200–202). Underlying these observations, in stem cells, the expression of Myc contributes to the high ribosome biogenesis when mTOR signaling is downregulated to maintain its pluripotency (203,204). Upon switching to the differentiation program, the resource shifts from ribosome biogenesis to protein synthesis to meet the demands of differentiation (191, 198, 199). Recent studies suggest that the reduction in rRNA synthesis actively induces stem cell differentiation in mammalian cells, challenging the notion that it is merely a consequence of differentiation (195). Given the important role of ribosome biogenesis in both stem cells and cancer, it prompts the intriguing question of whether cancer stemness relies on its support.

Tumors consist of diverse subpopulations of cells, encompassing both differentiated and stem-like cells. The minor subset of tumor cells exhibiting stem-like properties possesses the capacity for self-renewal and assumes a pivotal role in contributing to tumor heterogeneity, recurrence, and metastasis (205). For example, in colorectal cancer (CRC), increased rRNA production and protein synthesis have been found to be associated with cancer stemness, particularly in specific tumor niches adjacent to the stroma. Cells within these niches exhibit overexpression of RPA194 (POLR1A), resulting in increased rDNA transcription compared to normal cells, which correlates with poor survival in patients (206). Single-cell profiling pseudotime reconstruction, which analyzes the gene expression dynamics during the differentiation process, revealed that high-RPA194 cells serve as the origin of the tumor cell hierarchy. As cells differentiate, both rRNA synthesis and protein synthesis rates decline. Interestingly, this subset of RPA194-high CRC cells constitutes the majority of metastasis-initiating cells, displaying enhanced rDNA transcription and protein synthesis crucial for tumor growth. Moreover, genetic ablation of this high-RPA194 cell population is sufficient to irreversibly halt CRC growth, emphasizing the pivotal role of ribosome biogenesis in seeding cancer growth (206). These findings indicate a commonality between cancer cells acquiring stem-like properties and natural stem cells—both show heightened ribosome biogenesis, and the elevated protein synthesis rate aligns with that observed in stem cells during differentiation. This shared characteristic may underscore a fundamental mechanism supporting stemness features in cancer cells.

Beyond the core RNA Pol I machinery, various factors contribute to cancer stemness by influencing ribosome biogenesis. One noteworthy example is Ect2, an oncogenic guanine nucleotide exchange factor (GEF) overexpressed in diverse cancers. The expression level of Ect2 correlates with a transcriptional signature indicative of cancer stemness (207–209). In a KRAS-TRP53-driven lung adenocarcinoma model (LDAC), it has been demonstrated that the nuclear GEF activity of Ect2 is indispensable for maintaining tumor-initiating cells (TICs), and the transformative effects mediated by Ect2 rely on Ect2-associated rDNA transcription. Mechanistically, Ect2 activates rRNA synthesis by binding to UBF1 on rDNA promoters, recruiting Rac1, and engaging its downstream effector nucleophosmin at the rDNA site. Crucially, the interaction between Ect2 and UBF1 was specifically detected in LDAC cells, distinguishing it from non-transformed lung epithelial cells. In the absence of nuclear Ect2 activity, LDAC TICs are unable to reconstitute tumor growth, emphasizing the essential role of Ect2 in promoting rRNA synthesis—a prerequisite for sustaining stem-like TICs in LADC—and underscoring the involvement of ribosome biogenesis in cancer stemness (208,209).

**Figure 3.**
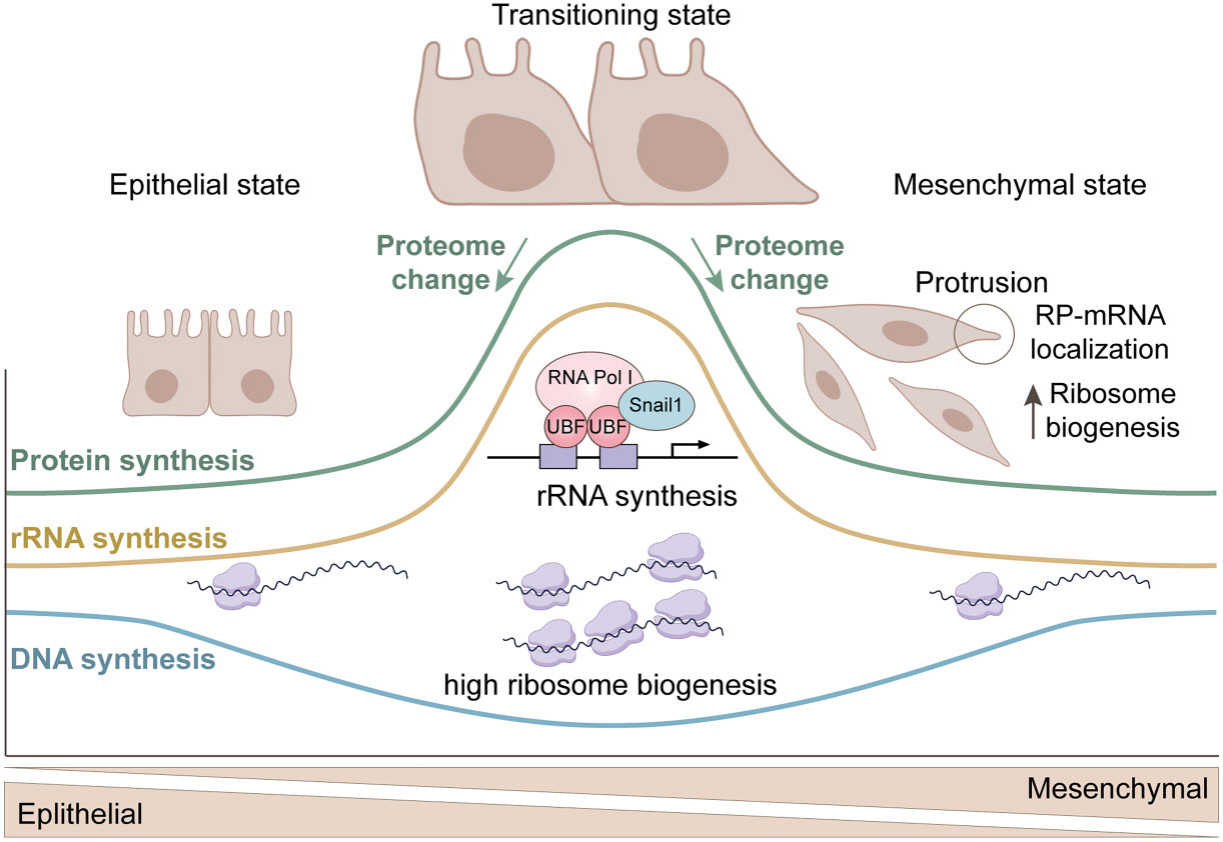
The interplay between cell proliferation, rDNA transcription and protein synthesis during EMT and MET. During EMT, along with cell cycle arrest and reduction in DNA synthesis, there is a surge in rRNA production coupled with the enlargement of nucleoli, indicating an activation of ribosome biogenesis. The elevated ribosome level is essential to meet the high demand for protein synthesis, facilitating the establishment of a new proteome during and after the transition. Similarly, this trend is mirrored in the reversal process, MET, underlining the critical role of increased ribosome biogenesis and protein synthesis during these transition processes. Additionally, in migrating mesenchymal cells, there's a specific localization of RP-mRNAs translation within cell protrusions, contributing to an overall increase in ribosome biogenesis required for the cell invasion process. Created with BioRender.com.

Recent studies unveiled that the EMT process also demands precise control of rDNA transcription, indicating a potential link between the molecular mechanisms guiding EMT and the regulation of ribosome biogenesis (Figure 3). Integral to metastasis and cancer progression, EMT is closely associated with the acquisition of stemness characteristics. During EMT, cancer cells undergo a transformation where epithelial cells de-differentiate, acquiring mesenchymal properties akin to stem cells (210). This alteration empowers the cells with migratory abilities, allowing them to disseminate from primary tumors to distant sites, instigating invasion and metastasis. This transition not only contributes to tumor heterogeneity and metastasis but also confers therapy resistance (210–212).

While ribosome biogenesis is dynamically regulated throughout the cell cycle, with high activity associated with cell proliferation, an augmentation in rRNA synthesis during EMT has been shown to occur during the G1/S cell cycle arrest that accompanies both the initiation and execution of EMT (213). This is supported by the observed enlargement of nucleoli, alongside elevated expression of Pol I machinery components and factors involved in rRNA processing and ribosome maturation, collectively indicating the activation of ribosome biogenesis. Remarkably, the EMT program triggers the activation of rDNA genes that are typically maintained in a silent state. Simultaneously occurring with the induction of the mesenchymal gene expression program and the emergence of a migratory phenotype, it has been demonstrated that this process involves the dissociation of the repressive nucleolar chromatin remodeling complex (NoRC) from rDNA. Additionally, an enhanced association of Pol I, UBF and the EMT-promoting transcription factor Snail1 with rDNA occurs during this phase. While the recruitment and the precise role of Snail1 in regulating rRNA synthesis are not well-defined, elevated Snail1 expression alone is sufficient to induce rRNA synthesis, even in the absence of an EMT inducer like TGFβ. Snail1's active involvement in rRNA transcription points to the essential requirement for increased ribosome biogenesis within the integral framework of the EMT program. Consequently, inhibiting ribosome biogenesis with a small-molecule inhibitor targeting the Pol I complex hinders the EMT program and diminishes the metastatic potential of primary tumors, as well as the seeding of metastasis in a mouse model of metastatic breast cancer (213).

Aiming to further dissect how ribosome biogenesis contributes to the transition between epithelial and mesenchymal states during metastasis, another study analyzed the transcriptome alterations using an EMT lineage-tracing assay in the MMTV-PyMT transgenic mouse model of breast cancer. This model expresses RFP during the epithelial state and progressively transitions to expressing GFP induced by mesenchymal markers during the shift to a mesenchymal state in metastasis, allowing precise analysis by single-cell sequencing. The analysis of cells at epithelial state, mesenchymal state and transitional state of EMT revealed a substantial activation trend in ribosome biogenesis during the transitioning phase, which was concomitant with an increase in nascent protein synthesis (214). This increase in ribosome biogenesis during the EMT transitioning phase was associated with dysregulated ERK and mTOR signaling pathways, which potentially support protein synthesis in tumors, facilitating the transitions of status (214). In metastasis, after cancer cells undergo EMT and disseminate from the primary tumor site, a reversed process, Mesenchymal-Epithelial Transition (MET), is required for the disseminated mesenchymal cancer cells to transform back to an epithelial state in order to colonize and proliferate in secondary sites. During this reversal process, the pattern of ribosome biogenesis mirrored that observed during EMT, with high ribosome biogenesis and protein synthesis levels correlating with the transitional status. Moreover, inhibiting ribosome biogenesis, either by Pol I inhibitors or depleting ribosomal proteins, hindered both the EMT and MET processed in tumor cells. Together, these indicate a necessity for elevated ribosome biogenesis in both EMT and MET directions and suggest ribosome biogenesis is tightly regulated between the two essential transition processes of metastasis (214).

How does the augmentation of ribosome biogenesis and protein synthesis contribute to the EMT and MET processes? The substantial increase in size observed in G1/S-arrested cells during the EMT process suggests that an upsurge in ribosome production may be necessary to scale up the machinery required for maintaining protein synthesis in proportion to the altered cell size (213). Similarly to the hypothesized role in stem cells (191), ribosome biogenesis may actively contribute to the proteome change associated with the execution of the EMT or MET programs, involving the transition between an epithelial and a mesenchymal cell fate, and vice versa.

Notably, a specific mode of ribosomal protein production significantly influences the EMT process. Ribosome profiling studies have demonstrated an upregulation in the translation of ribosomal proteins, along with many translational factors, during EMT in breast cancer (215). Moreover, a recent study using a combination of RNA-seq and RNA fluorescence *in situ* hybridization (RNA-FISH) revealed an enrichment of ribosomal protein-encoding mRNAs (RP-mRNA) at the protrusions of migrating cancer cells (216). This localization is facilitated by LARP6, an RNA-binding protein upregulated during EMT in aggressive breast carcinomas. Through binding to RP-mRNAs, LARP6 promotes their localization to the protrusive edge of migrating mesenchymal-like cells, enhancing their translation in a region enriched with active translation machinery. The newly translated ribosomal proteins are believed to be transported back to the nucleolus for assembly into ribosomal particles, thereby increasing ribosome biogenesis and the global translation rate. These findings imply that the precise localization of ribosomal protein transcripts plays a crucial role in migrating mesenchymal-like cancer cells. This may establish a feedforward mechanism, enabling these cells to produce larger quantities of ribosomes necessary to produce a proteome supporting the invasion process (216). These insights may have significant implications for understanding and targeting metastasis.

In summary, accumulating evidence underscores the significant role of ribosome biogenesis and protein synthesis not only in the initial growth and proliferation but also in various stages of cancer progression. The coordinated regulation between ribosome biogenesis and protein synthesis plays a pivotal role in critical steps of metastasis, encompassing the stem-like characteristics of TICs, the EMT process for acquiring stemness, and the capacity for colonization in secondary sites through the reverse process, MET. These discoveries illuminate novel avenues for cancer treatment, suggesting therapeutic strategies that involve targeted manipulation of ribosome biogenesis and protein synthesis to specifically address cancer-like stem cells implicated in metastasis and cancer recurrence.

## Cancer-driven changes in nucleolar structure and function

The nucleolus is a nuclear membrane-less organelle serving as the epicenter for coordinating ribosome biogenesis, with a structure dynamically organized through liquid-liquid phase separation (LLPS) (217). Central to this orchestration is the active transcription of the pre-rRNAs by RNA Pol I and the recruitment of the rRNA processing complexes that initiate self-assembly of the nucleolus around the tandem arrays of rDNA repeats, termed nucleolar organizer regions (NORs) (218,219). Within the human genome, over 400 copies of rDNA are distributed along five chromosomes, with interspersed active and inactive gene repeats. Notably, only a subset of these rDNA genes remains actively engaged in ribosome synthesis (220).

The nucleoli of mammalian cells assemble with a distinctive internal architecture comprising three phase-separated subcompartments, each associated with specific stages of ribosome production: the fibrillar center (FC), the dense fibrillar component (DFC), and the granular component (GC) (217) (Figure 1). Within this intricate arrangement, the FC and DFC assemble as functional units, existing in multiple copies enveloped within a singular GC. The FC-DFC modules are where Pol I complexes concentrate to host the transcriptionally active rDNAs gene, producing the pre-rRNA that will be subjected to the initial ribosomal subunit maturation steps. The GC serves as the site for late-stage pre-ribosome maturation before their release into the nucleoplasm. A typical human nucleolus comprises several dozen FC-DFC modules, and their quantity is consistently increased in cancer cell lines. The liquid phase separation within the nucleolus could be potentially altered in cancer to a more liquid state, increasing the speed of rRNA processing, assembly and export (217,221). More studies are needed to determine whether a shift in liquid properties contributes to the elevated ribosomal activity observed in many cancer cells and how this could be harnessed as a therapeutic approach.

Consistent with an increased rate in ribosome production, the nucleolus undergoes significant alterations in size and structure during various stages of cancer development. These changes are routinely examined by pathologists as part of diagnostic assessments (222,223). One prominent morphological change observed in cancer cells is nucleolar hypertrophy, characterized by an increase in nucleolar size. In multiple cancer cell lines, this phenomenon is closely associated with heightened transcriptional synthesis of rRNAs. The visualization of nucleolar organizer regions in tumor tissues using the silver staining-based cytochemical technique AgNOR (silver-associated nucleolar organizer regions) (224,225), revealed that nucleolar hypertrophy correlates with enhanced RNA Pol I transcription, reflecting the increased demand for ribosome production to meet the heightened metabolic requirements of tumor progression (222,226). Nucleolar morphological changes serve as diagnostic indicators for pathologists assessing cancer biopsies. The evaluation of nucleolar size, along with other cellular features, contributes to the determination of the cancer's grade, aggressiveness, and potential response to treatment. Nucleolar hypertrophy, in particular, is often associated with high-grade tumors, indicating a more aggressive and rapidly proliferating cancer phenotype (222,227–229).

The nucleolus also serves as a central hub for stress sensing and its integrity relies on the precise coordination of rRNA transcription, processing and assembly with ribosomal proteins (230,231). Disruptions in these processes initiate nucleolar stress, characterized by G1 cell cycle arrest and/or apoptosis, contingent upon the stabilization of the tumor suppressor p53 (232–235). Activation of p53 constitutes a fundamental response mechanism triggered by various stresses or loss of cellular homeostasis (reviewed in (84)). Ribosomal proteins play a pivotal role in mediating the p53 signaling response following nucleolar stress.

Upon disruption of rRNA synthesis, one of the primary mechanisms activating p53 involves the binding of unassembled 5S RNP to MDM2, the E3 ubiquitin-protein ligase that typically targets p53 for proteasomal degradation in the absence of cellular stresses. This interaction inhibits MDM2's E3 ligase activity, leading to the stabilization and accumulation of p53 in the nucleus, where it upregulates target genes associated with cell cycle arrest and apoptosis (48,49,236,237). The p53-driven transcriptional program and its subsequent cellular outcomes, whether survival or cell death, exhibit tissue-specific variations and depend on the encountered stress type. Investigations into the consequences of nucleolar stress, particularly after inhibiting rRNA synthesis in cells derived from solid tumors, have unveiled varying degrees of sensitivity (233,238–240). In these cases, cytostatic effects, characterized by a G1-cell cycle arrest with minimal cell death, are observed. These effects are predominantly attributed to the transcriptional activation of p21^CIP1^ (234,241,242).

Given the escalating interest in targeting ribosome synthesis as a novel cancer therapeutic approach, understanding the p53-specific transcriptional response to nucleolar stress has become paramount. Gene expression studies in cancer cells with active p53 reveal that inhibiting ribosome biogenesis activates a p53-dependent transcriptional survival program (243,244). This program includes genes associated with autophagy induction, metabolism, and ROS control, which may explain why certain tumors stop dividing but do not undergo apoptosis after ribosome biogenesis inhibition (243–245). While mutations in the p53 gene are prevalent in cancer, certain cancer types, such as cervical and thyroid cancers, exhibit a significant proportion of tumors retaining the wild-type (WT) p53 (246, 247, 56, 248, 249). Moreover, the majority of p53 mutations are missense mutations, resulting in the production of full-length proteins that are stably expressed in tumor cells. The persistence of mutant p53 in cancer implies a selective advantage, as it has been demonstrated to support proliferation, metastasis, and confer drug resistance (250,251). Mounting evidence suggests that akin to WT p53, mutant forms can respond to cellular stresses associated with tumorigenesis and orchestrate transcriptional adaptive responses that promote tumor progression, including metabolic rewiring (252–254). Autophagy has emerged as a major metabolic regulator of cancer therapy resistance in different cancer type expressing either wild-type or mutant p53 (255,256). However, it remains to be elucidated whether mutant p53 directly regulates an autophagy transcriptional response involved in drug resistance. Studies examining the response of various p53 mutants to ribosome biogenesis inhibition are crucial for understanding their effects and to design more effective synergistic approaches with cytotoxic rather than cytostatic effects, minimizing the risks of therapy resistance. Nevertheless, targeting autophagy pathways may enhance the efficacy of ribosome biogenesis drugs in cancers with active p53, a topic explored further in the subsequent section.

## Targeting ribosome biogenesis for cancer therapeutics

Given its pivotal role in cancer growth, proliferation and progression, ribosome biogenesis emerges as a promising target for cancer therapy. Inhibiting ribosome biogenesis offers several advantages in cancer treatment. Firstly, cancer cells exhibit an elevated rate of ribosome biogenesis compared to normal cells, enhancing the specificity of drugs targeting this process to cancer cells. Secondly, inhibiting ribosome biogenesis holds the potential to hinder cancer recurrence and metastasis. As discussed earlier, emerging evidence indicates that the increased rate of ribosome biogenesis is not only crucial for primary tumor growth but also plays a pivotal role in metastasis and recurrence events, such as the EMT/MET process and the acquisition of cancer stemness (206,213,214).

In addition to exhibiting a higher rate of rRNA synthesis, cancer cells are also more sensitive than normal cells to inhibition of rRNA production (reviewed in (257–260)). For this reason, there is a growing interest in developing novel targeting approaches to inhibit rRNA transcription for cancer therapeutics. While several non-specific drugs, such as Oxaliplatin and Actinomycin D (which has demonstrated preferential inhibition of RNA Pol I over RNA Pol II at low concentrations), are currently utilized in cancer therapy (261,262), there is a dedicated focus on creating selective inhibitors of the RNA Pol I machinery. The aim is 2-fold: (a) to develop a non-genotoxic treatment that does not impact the DNA/genetic information of healthy cells and (b) to evaluate the therapeutic potential of inhibiting rRNA transcription by gauging the extent of therapeutic efficacy solely by targeting this process while sparing other RNA polymerases. The promise of such treatment lies in its ability to potentially address a wide array of cancers, given the commonality of rRNA upregulation/dysregulation in most, if not all, cancers. Moreover, the selectivity towards malignant cells is maintained, as the level of rRNA transcription in healthy cells remains relatively low. Much of the focus for developing selective inhibitors for rRNA synthesis has been on blocking or disassembling the Pol I transcription machinery. Here, we highlight select compounds that have demonstrated efficacy in inhibiting ribosome biogenesis. For a comprehensive review of various ribosome biogenesis inhibitors identified, we refer to (263).

BMH-21, an acridine-like quinazolinone derivative, exhibits selective intercalation into GC-rich DNA sequences (264). Originally identified through anticancer agent screening, BMH-21 has been demonstrated to inhibit Pol I transcription by inducing the proteasomal degradation of the RPA194 subunit of Pol I, consequently impeding elongation. This inhibition is achieved by reducing Pol I occupancy and increasing pauses on rDNA (265–267). Although BMH-21 activates p53, it distinguishes itself from many ribosome biogenesis inhibitors that act as DNA intercalators by not inducing DNA damage. The primary mechanism of action involves the intercalation of BMH-21 in rDNA GC-rich sequences, resulting in the inhibition of transcription initiation and elongation. BMH-21 has proven effective in inhibiting the replication and viability of a diverse range of cancer cell lines, exhibiting significant repression of tumor growth of A375 melanoma xenografts *in vivo* (268).

Recently, a synergistic approach involving the combination of BMH-21 with the chemotherapy drug cyclophosphamide (CTX) has demonstrated a remarkable reduction in breast cancer metastasis, both *in vitro* and *in vivo*. Notably, CTX alone was effective in eliminating the majority of tumor cells, except for a small subset that underwent EMT, acquiring mesenchymal properties and chemoresistance. However, the introduction of BMH21, inhibiting ribosome biogenesis, counteracted chemoresistance by impeding the EMT/MET process when combined with CTX. This observation emphasizes the effectiveness of inhibiting ribosome biogenesis as a strategic approach to mitigate chemoresistance in cancer therapy (214).

BMH-9, BMH-22 and BMH-23 have been identified as compounds capable of inducing RPA194 degradation, leading to the inhibition of rRNA synthesis. Preliminary assessments of BMH-22 in a B cell lymphoma mouse model demonstrated anti-tumorigenic potential, and all three molecules exhibited growth inhibitory activity in NCI60 cancer cell lines. BMH-9 and BMH-22 showed better tolerance in normal, healthy cells, whereas BMH-23 displayed more toxicity. Despite the promising efficacy of these RPA194-degrading molecules, none of them has advanced into clinical trials thus far (269).

CX-5461 (pidnarulex) is a selective Pol I transcription inhibitor displaying therapeutic potential in pre-clinical models of various cancers, including breast cancer (270), melanoma, pancreatic cancer (271), ovarian cancer (272), multiple myeloma (273), lymphoma (7), and prostate cancer (274). CX-5461 has successfully completed phase I clinical trials in patients with advanced hematologic and breast cancers (275) and the drug is advancing to phase Ib to establish a tolerable dose in selected solid tumors, including those with BRCA1/2, PALB2 or HRD mutations (NCT04890613). A phase I REPAIR trial, combining CX-5461 and the PARP inhibitor talazoparib, is underway to treat patients with metastatic castration-resistant prostate cancer (mCRPC) (ClinicalTrials.gov Identifier: NCT05425862). Initially recognized for its specific blockade of Pol I transcription by disrupting SL1-rDNA association (271), subsequent studies have unveiled that CX-5461 binds to and stabilizes G-quadruplex DNA structures, impeding replication forks and inducing DNA breaks (270,276,277). Although studies propose that the primary mechanism of CX-5461 involves topoisomerase II poisoning (276), a recent study suggested that CX-5461 primarily targets the initiation of Pol I by irreversibly inhibiting the release of RRN3 from the pre-initiation complex on the rDNA promoter. Importantly, this inhibition does not affect the subsequent elongation process (278). Further research is necessary to determine the specific molecular mechanisms underlying CX-5461′s activity, as multiple mechanisms may be at play. It is noteworthy that CX-5461 has been shown to causes extensive and nonselective mutation in *BRCA1-/BRCA2*-deficient cells as well as in normal cells, warranting caution for its use in human patients (279).

The majority of the aforementioned inhibitors exert their effects by intercalating into DNA, a mechanism that may induce DNA breaks and compromise genome integrity. This raises concerns regarding potential toxicity, as exemplified by the recently revealed mutagenicity of CX-5461. Consequently, there has been a quest to identify compounds capable of avoiding these deleterious effects. Recent discoveries have brought forth Metarrestin, a compound that disrupts the nucleolar structure, impedes RNA Pol I occupancy at rDNA, and inhibits its transcription—all without causing damage to DNA or inducing apoptosis (280). Notably, Metarrestin targets the perinucleolar compartment, rich in non-coding RNAs and RNA-binding proteins, which is more prevalent in metastatic cancer cells. While Metarrestin displayed only a modest effect on primary tumor growth, it significantly enhanced survival rates in metastatic pancreatic cancer. Moreover, its efficacy extended to reducing metastatic burden in various human cancer xenograft mouse models (280). These investigations mark the initial comprehensive endeavor to establish a link between the inhibition of ribosome biogenesis and cancer invasion and metastasis. Metarrestin is presently undergoing phase I clinical trials for the treatment of pancreatic cancer, breast cancer or solid tumors resistant to standard therapies (ClinicalTrials.gov Identifier: NCT04222413).

Several clinically approved drugs have demonstrated the ability to inhibit ribosome biogenesis, opening avenues for their repurposing in cancer treatment. A noteworthy example is the antimalarial drug amodiaquine, which triggers the degradation of the Pol I subunit RPA194, effectively suppressing rDNA transcription in a manner akin to BMH-21 (281). Amodiaquine exhibited notable cytotoxicity in diverse colon carcinoma cell lines, but its efficacy as an *in vivo* ribosome biogenesis inhibitor remains to be established. Importantly, amodiaquine also functions as an autophagy inhibitor (282). The dual action of inhibiting both ribosome biogenesis and autophagy could enhance its anti-tumor efficacy as autophagy emerges as a major mechanism of drug resistance (283) The repurposing of existing drugs with ribosome biogenesis inhibitory properties for cancer treatment holds the potential to expedite clinical trials and reduce developmental costs.

Despite being largely underexplored, rRNA processing factors also emerge as promising therapeutic targets, exemplified by the inhibitor SMIP34. This small peptide demonstrates its potential by targeting PELP1 and disrupting the assembly of the Rix1 complex, a crucial player in 60S ribosome maturation. Through direct binding to PELP1, SMIP34 hinders the formation of the Rix1 complex, leading to PELP1 proteasomal degradation. This cascades into the downregulation of key Rix1 components—WDR18, TEX10, LAS1L, and SENP3—resulting in a subsequent reduction in rRNA synthesis. (284). Early studies have showcased the efficacy of SMIP34 in reducing the progression of endometrial cancer and ER-positive breast cancer, highlighting the potential of exploring rRNA processing factors as valuable therapeutic targets (284–286).

## Future perspective and conclusion

The rate of RNA Pol I transcription is heightened across various tumor types, establishing an increase in ribosome biogenesis as a hallmark of human cancers. Despite the prevalence of this phenomenon, the molecular intricacies governing the entire process of rRNA synthesis in human cells have been underexplored, leading to the oversight of therapeutic interventions targeting hyperactivated ribosome production in cancer. Only in the last decade have efforts emerged to specifically address hyperactivated Pol I transcription using novel small molecule inhibitors. The design of drugs targeting ribosome biogenesis is an ongoing endeavor, showing significant promise as an emerging cancer therapeutic. The current inhibitors available primarily target the Pol I machinery; however, drugs like CX-5461 have recently been proposed to exert their cytotoxic effects by stabilizing G-quadruplex DNA structures and acting as topoisomerase II poisoning agents. Consequently, CX-5461 has demonstrated high mutagenicity (270,276,279). The DNA damage associated with these mechanisms of action raises caution in the application of these drugs. Moreover, these drugs may also exert some of their cytotoxic effect via non-nucleolar events, which warrant further investigations. Exploring downstream events of rRNA synthesis, such as processing and ribosome assembly, may also yield robust inhibition of cancer cell proliferation and warrants further investigation. Among various processing and assembly factors, numerous are enzymes such as ATPases, GTPases, and nucleases that possess catalytic pockets suitable for designing small molecules to target their function, ensuring feasibility and potential specificity in interventions. Recent pan-cancer analyses across 20 human cancers have revealed that many of these rRNA processing and ribosome assembly factors are overexpressed in human cancers, underscoring their clinical relevance and the pressing need to better understand their functions in cancer (140). Recent studies in yeast have identified promising inhibitors like Diazaborine and Ribozinoindoles, which target the ATPases Drg1 and MND1, respectively. These inhibitors, involved in downstream steps of ribosome maturation, have demonstrated efficacy in inhibiting cell growth and proliferation (287,288). While promising, it remains to be determined whether these inhibitors will prove efficacious in inhibiting the proliferation and tumorigenesis of human cells. Future studies aimed at unraveling the deregulation of the 200-plus accessory factors involved in rRNA synthesis and ribosome assembly in human cells will enhance our understanding of the key events driving increased rRNA production in cancers, revealing new potential druggable targets.

Traditionally perceived as uniform molecular machines with constitutive roles in mRNA translation, ribosomes have recently been discovered to exhibit unexpected heterogeneity, with diverse subpopulations capable of selectively modulating translational programs based on cellular context (181). This heterogeneity, stemming from variations in ribosomal protein composition (this topic is explored further in the review from William Faller in this collection) and the presence of rRNA modifications (this topic is explored further in the review from Jean-Jacques Diaz in this collection), allows ribosomes to differentially translate specific transcript sub-pools involved in processes like cell cycle regulation and development (130–135,181,289). While this review primarily focuses into the mechanisms and consequences of increased ribosome biogenesis in cancer, the intertwining regulatory mechanisms of ribosome biogenesis and heterogeneity raise questions about their interplay and significance in cancer progression. Given that most rRNA processing and modifications occur concomitantly with transcription, the factors involved likely exhibit tight spatial and temporal coordination with RNA Pol I elongation (12,98,99). This implies that changes in RNA Pol I transcription rate could result in alteration in rRNA modification pattern. However, it remains unclear whether the observed increase in Pol I activity in cancer drives alterations in modification patterns or if additional coordinating mechanisms exist. Factors like Myc, known to boost rRNA transcription, also enhance the transcription of snoRNAs and the rRNA methyltransferase fibrillarin, influencing rRNA modifications (290). Yet, the mechanisms and factors orchestrating this coordination, particularly those modulating RNA Pol I progression to regulate transcription elongation rates and coordinate rRNA modification, remain elusive. Nonetheless, this coordinated strategy likely allows cancer cells to exploit variations in both ribosomal quantity and quality, optimizing translation to promote their progression.

Research has predominantly focused on the correlation between ribosomal heterogeneity and differential translation in cancer development (291,292) while the influence of ribosomal production levels on this process has been relatively underexplored. This prompts the question: does high ribosome biogenesis rates result in an overall increase in global translation? On an intuitive level, an elevated ribosome production should be linked to an augmented translation capacity, thereby increasing global translation. In studies involving the Myc-induced lymphoma model, it was found that Myc hyperactivation leads to an abnormal increase in cap-dependent translation at the expense of internal ribosomal entry site (IRES)-dependent translation initiation of critical mitotic regulators. This dysregulation contributes to genomic instability (6). Although Myc's impact on translation can occur through simultaneous mechanisms, this implies that an increase in ribosome production may be associated with a boost in global translation.

Conversely, the effect of reduced ribosome levels in decreasing the global translation rate is well demonstrated in ribosomopathies (168–170). In Diamond–Blackfan anemia (DBA), about 50% of patients exhibit loss-of-function mutations in ribosomal proteins. Despite a reduced global translation rate, specific mRNAs, such as GATA1, crucial for erythroid development, are highly sensitive to changes in ribosome levels. (182,183). Furthermore, even with no change in ribosome composition, a decrease in ribosomal levels can differentially affect the translation of various subsets of mRNA (183). These findings suggest that alterations in ribosome biogenesis can have a substantial impact on differential translation, even in the absence of other molecular mechanisms. This is primarily due to the varying sensitivity of certain mRNA to changes in ribosome levels. Together, ribosome biogenesis not only increases protein synthesis capacity but also refines the translational landscape to promote cancer progression.

More analyses are needed to unravel the implications underlying the effects of ribosome biogenesis on altering translation in cancer, as well as how it collaborates or influences ribosome heterogeneity to impact translation in a synergistic manner. Combining ribosome profiling and quantitative proteomics is crucial to evaluate the collective impact of increased global rRNA synthesis and modifications on the tumor proteome, elucidating their potential pivotal roles in cancer development (this topic is explored further in the review from Angel Roman in this collection). Establishing links between altered ribosome production, modification patterns, and translation signatures will reveal another level of tumor gene expression regulation, aiding in tailoring therapeutic strategies.

## Data Availability

No new data were generated or analysed.
